# Phylogeny of the infectious hematopoietic necrosis virus in European aquaculture

**DOI:** 10.1371/journal.pone.0184490

**Published:** 2017-09-08

**Authors:** Michael Cieslak, Thomas Wahli, Nicolas Diserens, Olga L. M. Haenen, Heike Schütze

**Affiliations:** 1 Institute of Infectology (IMED) of the Friedrich-Loeffler-Institut (FLI), Federal Research Institute for Animal Health, Insel Riems, Germany; 2 Centre for Fish and Wildlife Health (FIWI), Vetsuisse-Faculty, University of Bern, Bern, Switzerland; 3 Wageningen Bioveterinary Research, National Reference Laboratory (NRL) for Fish, Shellfish, and Crustacean Diseases, Lelystad, the Netherlands; INRA, FRANCE

## Abstract

Infectious hematopoietic necrosis (IHN)—a highly lethal infectious salmonid disease—has caused substantial economic losses in the European production of rainbow trout (*Oncorhynchus mykiss*) since the late 1980s. The causal agent of IHN is the IHN virus (IHNV) introduced from overseas. However, until today, its phylogeographic spread in Europe remains poorly understood. We therefore sought to elucidate this unresolved topic by using the largest ever compiled dataset of European IHNV isolates (*E* isolates) (193 GenBank *E* isolates and 100 isolates from this study) for the complete glycoprotein (*G*) gene sequence. Our results clearly revealed that the active trout trade has left its traces in the *E* phylogeny. For example, the spread by trade of IHNV-infected trout was apparently the cause for the exposure of the *E* lineage to different local scenarios of selection and genetic drift, and therefore has led to the split of this lineage into various subordinated lineages. Accordingly, we also found evidence for *E* isolates being mixed Europe-wide by cross-border introduction events. Moreover, there were indications that this propagation of the *E* lineage within Europe corresponded with an extensive and rapid spread event, already during or shortly after its formation. Finally, in accordance with the high substitution rate of IHNV determined by previous studies, our dataset indicates that the mean period of occurrence of a single *E* haplotype is typically not longer than one calendar year.

## Introduction

The economic efficiency of European rainbow trout (*Oncorhynchus mykiss*) aquaculture is threatened by a highly lethal fish disease, infectious hematopoietic necrosis (IHN) [1], among many other diseases [1]. The causal agent of IHN is infectious hematopoietic necrosis virus (IHNV). Substantial annual economic losses are incurred due to the loss of fish and the expense of disease control measures [2].

IHNV is an enveloped single-stranded, negative-sense RNA virus of the genus *Novirhabdovirus* in the family *Rhabdoviridae* [3]. The genome of this virus contains approximately 11,100 nucleotides and consists of six genes encoding a non-structural protein (NV) and five structural proteins: nucleoprotein (N), phosphoprotein (P), matrix protein (M), glycoprotein (G), and RNA polymerase (L) in the following arrangement: 3ʹ–N–P–M–G–NV–L–5ʹ [4–6].

The IHN disease is primarily transmitted from fish to fish, as well as by virus-contaminated water or objects [2]. Typical disease signs are lethargic behavior, darkening of the skin, distended abdomen, exophthalmia, pale gills, and petechial hemorrhaging. The mortality in IHNV-infected salmonid fish can be as high as 90%, especially in young fish [2]. Furthermore, adult fish can have high viral loads at or near the time of spawning [7].

IHNV has been found in North America, Asia and Europe, but not in the Southern Hemisphere. Countries reporting confirmed or suspect cases of IHN to the OIE include: Austria, Belgium, Canada, China (People’s Rep. of), Croatia, Czech Republic, France, Germany, Iran, Italy, Japan, Korea (Rep. of), the Netherlands, Poland, Russia, Slovenia, Spain, Switzerland and United States of America [2]. However, the ancestral lineage of the current IHNV isolates is assumed to have been originally endemic to the Pacific Coast of North America. This assumption is based on phylogeographic studies [8], but also on the fact that IHN outbreaks were restricted to this area until as late as the late 1960s. The first records of a salmonid disease exhibiting symptoms similar to those of the current IHN disease in this area are from the 1940s [9, 10]. In 1968, IHN was documented in Japan [11], and in 1987 in Europe [12, 13]. Shortly after this, IHNV appeared also in China in 1988 [14], in Korea in 1991 [15], and sporadic occurrences of IHN have also been reported in Russia (near Moscow in 2000 and in the Russian Far East in 2001) [16] and in Iran (in 2004) [17]. Currently, it is assumed that all these spread events were mainly the result of careless trade practices of IHNV-infected eggs or fry of salmonids [1].

In North America and Asia IHN has affected culture facilities of several salmon species, partly also with high mortalities, for example, of sockeye (*Oncorhynchus nerka*) [18], Atlantic salmon (*Salmo salar*) [19], rainbow trout [20], and masu salmon (*Oncorhynchus masou*) [21]. In European countries, however, IHN has had its major adverse effect on rainbow trout production. As aquaculture of salmonid fish is an industry of major economic importance worldwide, IHN has been categorized a notifiable disease by the OIE (World Organization for Animal Health) [2] and European Union [22].

Phylogenetic analyses have classified IHNV isolates into five genogroups, namely, *U*, *M*, *L*, *J*, and *E* [8]. The phylogenetic division into *U*, *M*, and *L* is based on the original North American geographic distribution of IHNV along the Pacific Coast (*L*: lower region; *M*: middle region; and *U*: upper region) [23]. It was estimated that the most recent common ancestor of these three genogroups has lived during the early 1950s [8]. In the mean time, the *U* isolates have also been found in Japan and the Russian Far East [16], whereas *M* isolates have also been found in China [14]. *J* isolates, on the other hand, have only been found in Asia (Japan, China, and Korea) [8, 14, 15], and *E* isolates have only been found in Europe [24] and in Iran [17].

It is assumed that the European *E* lineage has separated from the *M* linage during the 1970s, although the first European evidence of IHN was in 1987 [8]. Enzmann and his colleagues [24] have divided the *E* lineage phylogenetically into seven subgenogroups (*A–G*). However, to this day, the conclusive clarification of the phylogenetic relationship between these groups has remained largely impossible. This is due to many low bootstrap confidence values in the *E* phylogeny as seen in previous studies [24, 25]. Therefore, their individual spread routes have also remained largely unknown.

This study was carried out within the scope of a multidisciplinary trans-European research project, MOLTRAQ (molecular tracing of viral pathogens in aquaculture). We sought to shed new light on the geographic spread route of the *E* lineage in Europe, as such epidemiological knowledge can be a basic prerequisite for developing and implementing efficient IHN prevention and eradication measures in the future. For example, detailed information about a spread route can be helpful for the identification of its underlying causal risk factor, which then can be eliminated. In this study, we followed a phylogeographic approach in order to reveal spread routes of the *E* lineage. This means that we have combined information of (1) the phylogenetic radiation of the European IHNV lineage (*E* genogroup) into subordinated lineages, and (2) the country-specific distribution of each of these subordinated lineages.

Finally, we also used both a phylogenetic tree and a network method, although gene phylogenies of a virus are usually represented only by a rooted bifurcating tree for simplicity. This simplification, however, can imply the risk of unresolved conflicting branching patterns, as a bifurcation algorithm does not permit the correct phylogenetic resolution of a hard polytomy (a multiple, simultaneous divergence event) [26, 27]. At first sight, this might not seem that problematic, as researchers traditionally assumed that hard polytomies were exceptions and rare in nature. However, we think it is important to pay more attention to polytomies, especially when investigating spread routes of an agent with a high substitution rate and whose host is a frequently traded farmed species. Our underlying idea is that even a single delivery of IHNV-infected trout can cause a hard polytomy, as it can lead to the spread of one IHNV haplotype (or very closely related haplotypes) to various European regions at the same time.

## Material and methods

### Ethics statement

Ethical approval was not required for this study. IHNV samples were obtained from European health services and regional laboratories and were isolated from fish on the basis of the Council Directive 2006/88/EC of the European Union (EU) on animal health requirements for aquaculture animals and products thereof, and on the prevention and control of certain diseases in aquatic animals [22].

### Collection of IHNV isolates

The viruses were sampled and isolated according to the standardized methods described in the Commission Decision 2001/183/EC of the EU [28]. We sampled 100 IHNV specimens: three from France, 76 from Germany, and 21 from Switzerland. These samples covered the period from 1993 to 2015 (S1 Table). Furthermore, 193 *G* gene sequences of European IHNV isolates from GenBank (National Center for Biotechnology Information; NCBI) were also incorporated into our dataset (one from Croatia, 19 from France, 65 from Germany, 92 from Italy, 12 from the Netherlands, four from Switzerland, and one from the USA, and they covered the period from 1982 to 2013) (listed in S1 Table and visualized in S1 Fig). The inclusion criteria were as follows: (1) the sequence of the complete *G* gene had to be listed; (2) the date of collection had to be recorded; and (3) the geographic site of collection had to be known.

### RNA extraction

Viral genomic RNA was extracted using the same method as previously described [26].

### RT-PCR and *G* gene sequencing

The RT-PCR and *G* gene sequencing was performed using the same methods as previously described [26]. Primers for RT-PCR were designed based on the published sequence of IHNV in the GenBank database under the accession number X89213 (S2 Table) [6]. The full-length G gene sequence was deposited in GenBank.

### Determination of the haplotype

The haplotype of a IHNV isolate was determined based on the substitution differences within the complete *G* gene sequence. In order to achieve this, a multiple sequence alignment was performed using Geneious Pro 7.1.7 Software (Biomatters Ltd.) [29]. In this context it is also important to mention that the evolution of IHNV may correspond to the quasispecies hypothesis [30, 31]. If this is the case, it can be assumed that an ancestral IHNV sequence changes rapidly into a ‘mutant cloud’ of closely related variants. By and large, we nevertheless expect a single sequencing signal of the *G* gene, as such a cloud is dominated by a master sequence that displays the highest replication rate due to its highest fitness among the variants. If specimens nevertheless featured a variation of two possible nucleotides at a single position of the *G* gene sequence, they were split into two haplotypes and phylogenetically handled as though they were two isolates. In case a specimen varied at more than one nucleotide position within the *G* gene sequence, they were excluded from the dataset. The total number of haplotypes was calculated using DnaSP version 5.10.01 [32].

### Phylogenetic subdivision of the *E* genogroup (*E* lineage)

Following the principle the lower the bootstrap support value, the less reliable the grouping, we divided the haplotypes of the *E* genogroup into *E* clades on the basis of a Maximum Likelihood (ML) bootstrap support value of >80%. These clades were, in turn, further divided into subordinated lineages on the basis of a Maximum Likelihood (ML) bootstrap support value of >50%. The bifurcating ML tree was constructed by the computer program MEGA version 5.2 [33]. The best-fit nucleotide substitution model was selected using the Bayesian Information Criterion score with Find Best DNA Model in MEGA version 5.2 [33]. As a result, the general time reversible (GTR) model with gamma rate heterogeneity and invariant sites was chosen, and 250 bootstrap replicates were generated to assess the reliability of the *E* subgenogroups obtained in the tree. Finally, the ML tree was transferred into a consensus tree, in which conflicting branching patterns are resolved by selecting the pattern seen in more than 50% of the trees. Furthermore, we created a phylogenetic Median Joining (MJ) network using the computer program NETWORK version 4.6.1.2 (http://www.fluxus-engineering.com) [34]. The program’s default setting of Epsilon (0) was chosen and the transition/transversion bias (R) was based on a maximum likelihood estimate obtained using MEGA version 5.2 [33]. The reliability of the MJ network topology was checked for its phylotemporal structure, and on the condition that each *E* haplotype of the MJ network is identically phylogenetically grouped in the ML tree.

### The mean period of occurrence in years of a *E* haplotype

The mean period of occurrence, in years, of a single *E* haplotype was calculated using the following criteria: 0, occurrence within one calendar year; 1, occurrence within two calendar years; and so forth.

### Phylogeographic analysis and nucleotide diversity

The phylogeography of the *E* population was illustrated by the MJ network constructed by NETWORK version 4.6.1.2 [34]. Nucleotide diversity (PI) was calculated using DnaSP version 5.10.01 [30].

## Results

We successfully sequenced and phylogenetically classified 100 IHNV specimens: three from France, 76 from Germany, and 21 from Switzerland. All these European isolates clustered within the European genogroup *E*. Their full-length *G* gene sequences were deposited in GenBank. In total (plus the GenBank isolates that were added to the dataset), the dataset comprises 294 isolates from Croatia (1), France (22), Germany (141), Italy (92), the Netherlands (12), Switzerland (25), and the USA (1), covering the period from 1982 to 2015 period (S1 Table and S1 Fig). Three German (LN897514, LN897545, and LN897570) and one Italian (KU878316) isolate featured a variation of two possible nucleotides at a single position of the *G* gene sequence. Therefore, they were split into two haplotypes and phylogenetically handled as though they were two isolates. Accordingly, our dataset includes four more isolates (298), which can be divided into a total of 216 haplotypes. When looking at the period each *E* haplotype occur, our dataset indicates that the mean period of occurrence is typically not longer than one calendar year.

**Fig 1:**
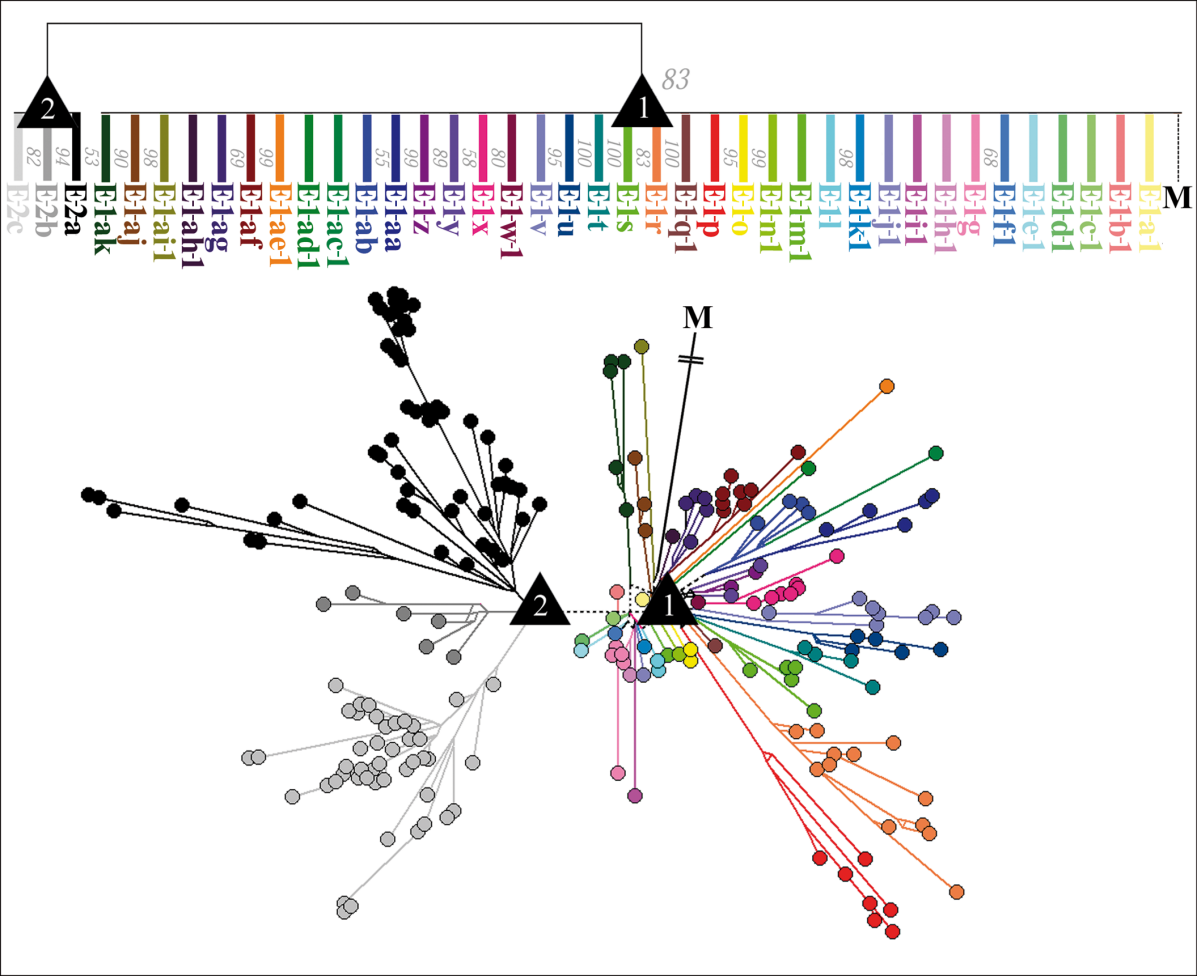
Consensus Maximum Likelihood tree and Median-joining network (transition/transversion bias = 4) of the *E* genogroup. The upper and the lower phylogeny show the phylogenetic relationship between *E* haplotypes of IHNV, based on the complete *G* gene sequence and were generated using 294 *E* isolates. The upper phylogeny is illustrated as a Consensus Maximum Likelihood tree (conflicting branching patterns are resolved by selecting the pattern seen in more than 50% of the trees). Numbers to the right of the branches represent the bootstrap support values obtained from 250 replicates. The lower phylogeny is illustrated as a Median-joining network. The subordinated lineages of clade *E*–*1* (*E–1–a* to *E–1–a–k*) and *E*–*2* (*E–2–a* to *E–1–c*) are indicated using a color code. Black triangles marked “1”or “2” represent nodes (node 1 and node 2) that correspond in the Median-joining network with a polytomy. Isolate *M* is the outgroup in each diagram.

In accordance with our above mentioned expectation to find polytomic structures in the *E* phylogeny caused by the active trout trade, we finally converted the ML tree into a 50% majority-rule consensus tree (Fig 1). Thus, any conflicting branching pattern (caused by e.g. a hard or soft polytomy) was resolved by forming a multifurcating branching pattern (a node with a polytomic structure). On the basis of this consensus tree, we divided the *E* haplotypes into two clades (*E*–*1* and *E*–*2*). Their ML bootstrap support value was 83%. We additionally subdivided the *E*–*1* haplotypes into 21 subclade lineages (*E–1–c*, *E–1–f*, *E–1–g*, *E–1–l*, *E–1–o*, *E–1–p*, *E–1–r*, *E–1–s*, *E–1–t*, *E–1–u*, *E–1–v*, *E–1–*w, E*–1–x*, *E–1–y*, *E–1–z*, *E–1–aa*, *E–1–ab*, *E–1–af*, *E–1–ag*, *E–1–aj*, and *E–1–ak*) and 16 single-haplotypes lineages (*E–1–a–1*, *E–1–b–1*, *E–1–d–1*, *E–1–e–1*, *E–1–h–1*, *E–1–i–1*, *E–1–j–1*, *E–1–k–1*, *E–1–m–1*, *E–1–n–1*, *E–1–q–1*, *E–1–ac–1*, *E–1–ad–1*, *E–1–ae–1*, *E–1–ah–1*, and *E–1–ai–1*), which showed no affiliation to any of these subclades. The *E1* ML bootstrap support values ranged from 55% to 100% (Fig 1). We also subdivided the *E*–*2* haplotypes into three subclades, whose bootstrap support values ranged from 53% to 94% (Fig 1).

This phylogenetic division by the consensus tree was identical to the phylogenetic grouping within the MJ phylogenetic network (Fig 1). Furthermore, in case of both phylogenies, node 1—the ancestral node (root) of all *E* lineages—and node 2—the ancestral node of all *E*–*2* lineages—corresponded to a polytomy. Especially node 1 appears in the form of a star-like phylogeny, as it was the origin of 21 *E*–*1* subclades lineages and of 16 *E*–*1* single-haplotype lineages, whereas node 2 was only the origin of three *E*–*2*-subclades lineages. In addition, node 1 was also the connection to the *M* lineage from overseas, and the origin of the E–2 lineage (Fig 1).

**Fig 2:**
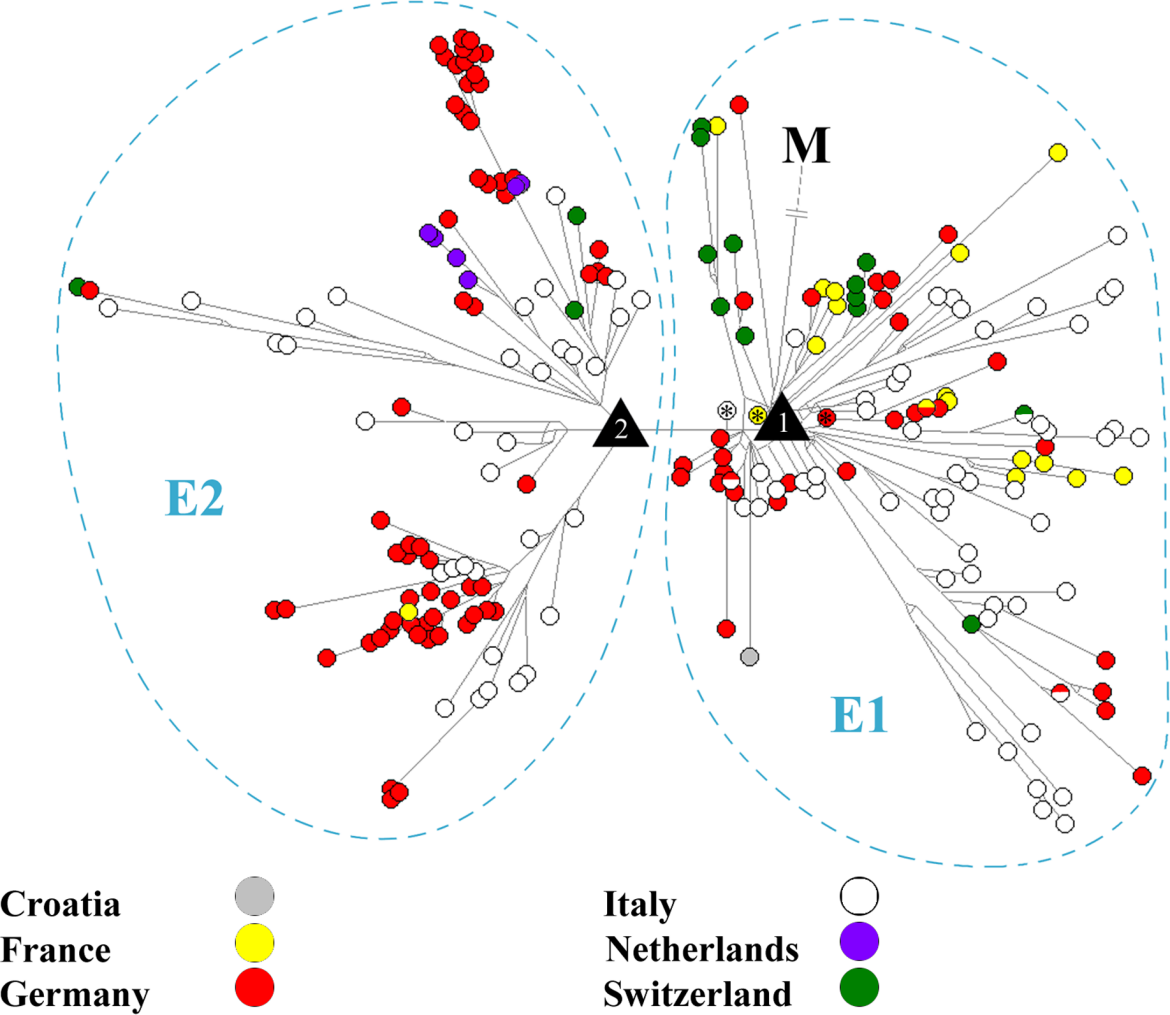
Median-joining network based on the complete *G* gene sequence from 294 IHNV isolates (transition/transversion bias = 4). The country is indicated using a color code. The black triangles with the number 1 and 2 represent polytomy nodes (node 1 and 2). In accordance with a largely phylotemporal structure in the network, the black triangle with the number 1 (node 1) is surrounded by the oldest French isolate (X89213 from 1987, marked by an asterisk), Italian isolate (FJ711518 from 1987, marked by an asterisk), and the second eldest German isolate (LN897500 from 1993, marked by an asterisk). The *E* haplotypes are divided into two clades (the *E1* and *E2* clade). Isolate *M* is the outgroup in the network.

In accordance with a phylotemporal arrangement of the *E* haplotypes in the MJ network (not shown), it was largely true that older isolates (earlier collection dates) clustered closer to the ancestral node of the lineage than isolates with more recent collection dates. For example, some of the first detected French, Italian, and German IHNV isolates (e.g. X89213, FJ711518, and AY331657) clustered tightly at the node 1 (Fig 2).

The nucleotide diversity (PI) of the total European *E* population (number of used sequences: 293) was 0.02745 PI. The country-specific nucleotide diversity was: 0.01896 PI for France; 0.02651 PI for Germany; 0.02664 PI for Italy; 0.00934 PI for the Netherlands; and 0.01993 PI for Switzerland.

On the basis of the phylogeographic pattern of the *E* lineage (Fig 2), no clear trend toward any European country was recognizable for the *E*–*1* isolates, nor for the *E*–*2* isolates. For example, the *E*–*1* isolates are from Croatia, France, Germany, Italy, and Switzerland, while the *E*–*2* isolates are from France, Germany, Italy, the Netherlands, and Switzerland (Fig 2 and S1 Table). In addition, four haplotypes of the *E*–*1* clade appear in more than one European country, the haplotype *E–1–v–2* (represented by the Italian isolate KU878281, and the Swiss isolates LN897488, LN897489, and LN897490), the haplotype *E–1–x–4* (represented by the German isolate EU676225, and the French isolates EU331445, EU331450, EU331451, and LN897477), the haplotype *E–1–r–11* (represented by the Italian isolates KU878359, KU878360, and KU878361, and the German isolates LN897531, LN897532, LN897533, and LN897535), and the haplotype *E–1–g–3* (represented by the German isolate EU676202, and the Italian isolate KU878286) (Fig 2). Nonetheless, in comparison to *E*–*1* isolates, *E*–*2* isolates clearly occur less often in France and Switzerland, whereas Dutch isolates all belong exclusively to the *E*–*2*–*a* subclade. Furthermore, there was also a country-specific occurrence for some subclades, for example, the Italian subclades *E–1–p*, *E–1–s*, and *E–1–t*, and the French subclade *E–1–u*.

## Discussion

Since the first *E* isolates of IHNV were detected in France and Italy in 1987, their descendants (and therefore also IHN) have subsequently spread in many other European countries during the last thirty years, most likely due to an active trout trade [e.g. 1, 24, 25, 35]. Therefore, it could be assumed that this trade-mediated spread has also led to the spread of IHNV-infected trout, resulting in the split of the *E* lineage into a variety of subordinated lineages caused by different local scenarios of selection and genetic drift [26]. Accordingly, our phylogenetic analyses revealed that the *E* lineage has undergone a split event (phylogenetic radiation) into two clade lineages (the *E*–*1* and *E*–*2* lineage), which in turn have been further split into several subordinated lineages (in case of the *E*–*1* lineage into 21 subclade lineages, and 16 single-haplotype lineages; in case of the *E*–*2* lineage into three subclade lineages) (Figs 1 and 2).

Furthermore, we also found evidence of this IHNV spread by trout trade on the basis of our phylogeographic analysis of the *E*–*1* and *E*–*2* clade, as it points to a Europe-wide mixing of *E* isolates. For example, the *E*–*1*, as well as the *E*–*2* population, did not show a clear country-specific distribution. Isolates of each clade were present in five out of six European countries of this dataset (Fig 2 and S1 Table). This is further underlined by the finding that isolates from Germany and Italy—the countries with the highest spatiotemporal sampling density in our dataset—(1) can each be found in almost 60% of all *E*–*1* subclade lineages, and even in 100% of all *E*–*2* subclade lineages. Furthermore, the value of the nucleotide diversity (PI) of the German and the Italian IHNV population was more or less similar to the value of the total European IHNV population. In addition, clear cross-border introduction events were also provided due to four types of *E*–*1* haplotypes, which we have found in more than one European country. We think that the reason why only four of such haplotypes were found goes back to the high mutation rate of IHNV [25]. This rate indicates a rapid sequence change of a *E* haplotype and therefore results in its short period of occurrence. This is also in accordance with our calculation that the mean period of occurrence of a single *E* haplotype is typically not longer than one calendar year.

At the first sight, the above mentioned Europe-wide mixing of *E* isolates by the active trout trade has largely led to an unstructured distribution of these isolates within Europe. There was no clear trend toward any European country for the *E*–*1* isolates, nor for the *E*–*2* isolates (Fig 2). Accordingly, we failed to infer a clear picture of a country-specific export or import trade of trout for most countries of this study. However, we nevertheless found some conspicuous patterns within the *E* phylogeography, which, at least to some extent, could point to a country-specific trade. For example, Dutch isolates are exclusively represented in the *E*–*2*–*a* subclade, and French and Swiss isolates occur less frequently in the *E*–*2* clade in comparison to the *E*–*1* clade. This could indicate a country-specific export and/or import trade of trout, in comparison to the German and Italian trout trade. Furthermore, the country-specific distribution for some sublcades (e.g. the Italian sublcaeds *E–1–p*, *E–1–s*, and *E–1–t*, and the French subclade *E–1–u*) may indicate a local absence of a cross-border export of IHN-infected trout. Nevertheless, these results must also be considered under the point of view that this study may represent an incomplete pan-European picture of the country-specific distribution for *E* isolates. This is because, although an extensive dataset of *E* isolates was used here, a spatiotemporal-sampling bias may exist. While Germany and Italy are represented by a particularly high spatiotemporal density of isolates in our dataset, such a sampling bias may exist in the case of *E* isolates obtained from the other countries (S1 Fig). This country-specific sampling is possibly based on, for example, outbreak samples from European trout farms (let alone from farms with latent infections) that were collected using different levels of strictness depending on individual national surveillance of IHN.

Moreover, the reconstruction of spread-routes from subordinated *E* lineages was obviously complicated due to: (1) this trout-trade-induced, strong Europe-wide mixing of *E* isolates, as it has led to barely identifiable traces of spread in the *E* phylogeography; and (2) the lack of a reliable clarification of the phylogenetic relationship between many *E* isolates, apparently because of two polytomic structures in the *E* phylogeny (node 1 and node 2). This is due to the fact that any polytomy presents, at first, an analytical problem. On the one hand, it can reflect a hard polytomy, and thus mirror the actual phylogenetic relationship. But on the other hand, it can also reflect a soft polytomy which only mirrors an unresolved conflicting branching pattern, supplying no information on the phylogenetic relationship [26, 27]. However, the determination of a hard polytomy requires a complex phylogenetic analysis. For example, it can be based on a multiple independent gene approach [36]. Given the fact that our dataset is based on a single gene, such an approach was impossible and therefore no final conclusion could be drawn.

Nonetheless, we think that node 1 and node 2 each most likely reflects a multifurcating split as a result of a previous hard polytomy. On the one hand, this hypothesis was based on our initial assumption that the trade of IHN-infected trout facilitates the occurrence of a polytomy, and therefore leads to such a rapid phylogenetic radiation event [26]. The underlying idea is that a single delivery of IHNV-infected trout can lead to the spread of one IHNV haplotype (or very closely related haplotypes) to various European regions at the same time. On the other hand, we found further indicators for a hard polytomy: (1) isolates clustering closest to node 1 (the ancestral node of the *E* lineage) were from different European countries (e.g. France, Germany, Italy, and Switzerland), and (2) node 1 was surrounded by the oldest French isolate (X89213 from 1987), the oldest Italian isolate (FJ711518 from 1987), and the second eldest German isolate (LN897500 from 1993) in accordance with a phylotemporal arrangement. These findings may also indicate that the rapid radiation of the *E* lineage has already begun during or shortly after its formation (Fig 2). Furthermore, we think that this hard polytomy at such an early stage of the *E*-lineage formation could be also one reason why it is particularly difficult to reconstruct its initial spread routes, let alone to define its geographic place of origin. This is based on the assumption that it must be held that a phylogeographic pattern is much easier to interpret, when, for example, a haplotype lineage has spread step by step from one country to another, as opposed to a haplotype lineage which has spread to several countries at the same time.

Finally, we believe that these polytomies node 1 and node 2 could also explain why up until now, previous studies largely failed to infer reliable intra-European spread routes on the basis of a bifurcating *E* phylogeny [e.g. 24, 25]. Usually, a reliable reconstruction of spread routes requires a robust phylogeny. However, as mentioned before, a bifurcation algorithm does not permit the correct phylogenetic resolution of a polytomy, which is why it often results in a low phylogenetic robustness.

In summary, our study revealed that analyses of individual spread routes for *E* isolates are particularly complicated, as the active trout trade has apparently left a hard-to-interpret phylogeographic pattern in the *E* lineage. In order to reconstruct reliable spread routes, we therefore recommend for future investigations: (1) a particularly deep spatiotemporal sampling of *E* isolates to overcome a bias; (2) the use of additional information regarding the commercial movement of the host species; (3) a genetic approach, for example, a multiple independent gene approach, which allows for the resolution and confirmation of a hard polytomy [36]; and (4) the use of a phylogenetic network method rather than a bifurcating method, as the algorithm of the latter does permit the correct phylogenetic resolution of a polytomy. Finally, we would generally recommend paying particular attention to hard polytomies in any species’ phylogeny, especially when its substitution rate is on a similar scale to IHNV and its host is a frequently traded farmed species.

## Supporting information

S1 Fig Chronology of the geographic collection of 293 European isolates. (TIF)

S1 Table Data on the 294 IHNV isolates used in this study. The samples on normal white background are the extended samples obtained from GenBank, whereas the samples highlighted in gray are the newly added samples from this study. The table includes the NCBI accession number, name of isolate, date of collection, site of collection, host species, and phylogenetic classification (genogroup-clade-subclade-haplotype). Samples with an underlined accession number are amplified and sequenced by Peter-Joachim Enzmann.
(DOCX)

S2 Table Primer sequences. Primers used for RT-PCR and sequencing of IHNV isolates in the present study.
(DOCX)
