# Supplementary figures and images for: Phylogeny of the infectious hematopoietic necrosis virus in European aquaculture
Source: PLoS One. 2017 Sep 8;12(9):e0184490. doi: 10.1371/journal.pone.0184490 (PMC5590938; doi:10.1371/journal.pone.0184490)

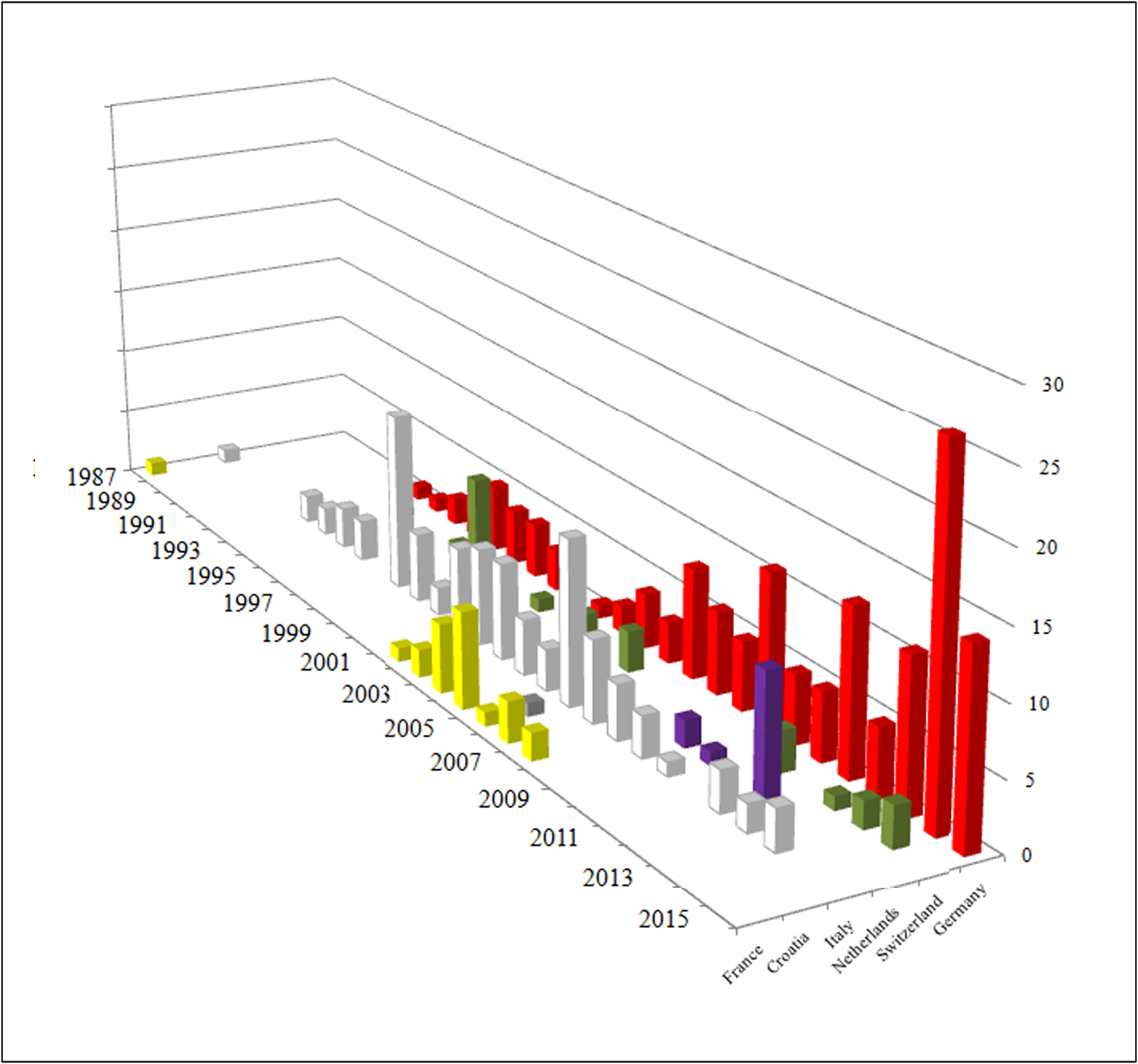

Supplement: S1 Fig — (TIF) [file pone.0184490.s001.tif]
