# Supplementary material for: Phylogeny of the infectious hematopoietic necrosis virus in European aquaculture
Source: PLoS One. 2017 Sep 8;12(9):e0184490. doi: 10.1371/journal.pone.0184490 (PMC5590938; doi:10.1371/journal.pone.0184490)
Supplement: S1 Table — The samples on normal white background are the extended samples obtained from GenBank, whereas the samples highlighted in gray are the newly added samples from this study. The table includes the NCBI accession number, name of isolate, date of collection, site of collection, host species, and phylogenetic classification (genogroup-clade-subclade-haplotype). Samples with an underlined accession number are amplified and sequenced by Peter-Joachim Enzmann. (DOCX) [file pone.0184490.s002.docx]

**S1 Table.** **Data on the 294 IHNV isolates used in this study.** The samples on normal white background are the extended samples obtained from GenBank, whereas the samples highlighted in gray are the newly added samples from this study. The table includes the NCBI accession number, name of isolate, date of collection, site of collection, host species, and phylogenetic classification (genogroup–clade–subclade–haplotype). Samples with an underlined accession number are amplified and sequenced by Peter-Joachim Enzmann.

| ***NCBI accession number*** | ***Name of isolate*** | ***Date of collection*** | ***Site of collection*** | ***Host species*** | ***Phylogenetic classification*** |
| --- | --- | --- | --- | --- | --- |
| X89213 | 1FO-87 | 1987 | France | Oncorhynchus mykiss | E–1–a–1 |
| EU331442 | F02001 | 2001 | France | Oncorhynchus mykiss | E–1–ag–1 |
| EU331443 | F92-02 | 2002 | France | Oncorhynchus mykiss | E–1–ag–4 |
| EU331447 | Fm13316-02 | 2002 | France | Oncorhynchus mykiss | E–1–u–1 |
| EU331446 | F28701-03 | 2003 | France | Oncorhynchus mykiss | E–1–ag–1 |
| EU331444 | F003454-03 | 2003 | France | Oncorhynchus mykiss | E–1–ad–1 |
| EU331448 | Fn6433-03 | 2003 | France | Oncorhynchus mykiss | E–1–u–3 |
| EU331449 | Fn11064-03 | 2003 | France | Oncorhynchus mykiss | E–1–u–2 |
| EU331452 | Fo43725-03 | 2003 | France | Oncorhynchus mykiss | E–1–x–5 |
| EU331453 | Fp7136-04 | 2004 | France | Oncorhynchus mykiss | E–1–u–4 |
| EU331454 | Fp7875-04 | 2004 | France | Oncorhynchus mykiss | E–1–ak–5 |
| EU331445 | F73801-04 | 2004 | France | Oncorhynchus mykiss | E–1–x–4 |
| EU331450 | Fn15209-04 | 2004 | France | Oncorhynchus mykiss | E–1–x–4 |
| EU331455 | Fp7946-04 | 2004 | France | Oncorhynchus mykiss | E–1–x–6 |
| EU331456 | Fsa041874-04 | 2004 | France | Oncorhynchus mykiss | E–1–ag–2 |
| EU331451 | Fn150974-04 | 2004 | France | Oncorhynchus mykiss | E–1–x–4 |
| EU676229 | F13849-05 | 2005 | France | Oncorhynchus mykiss | E–1–ak–5 |
| EU676237 | Fs360-06 | 2006 | France | Oncorhynchus mykiss | E–1–u–5 |
| LN897476 | F317630-06 | 2006 | France | Oncorhynchus mykiss | E–1–ag–3 |
| EU676230 | Dfr3164-06 | 2006 | France | Oncorhynchus mykiss | E–2–c–32 |
| LN897478 | E_Fft121-07iso | 2007 | France | Oncorhynchus mykiss | E–1–ae–1 |
| LN897477 | E_Fft121-07h | 2007 | France | Oncorhynchus mykiss | E–1–x–4 |
|  |  |  |  |  |  |
| EU219616 | Cro/05 | 2005 | Croatia | Oncorhynchus mykiss | E–1–i–1 |
|  |  |  |  |  |  |
| FJ711518 | 1IO | 1987 | Italy | Oncorhynchus mykiss | E–1–b–1 |
| KU878273 | IHNV/O.mykiss/I/TN/133/Jan92 | 1992 | Italy | Oncorhynchus mykiss | E–1–j–1 |
| KU878274 | IHNV/O.mykiss/I/TN/347/Mar92 | 1992 | Italy | Oncorhynchus mykiss | E–1–k–1 |
| KU878275 | IHNV/O.mykiss/I/TN/92/May93 | 1993 | Italy | Oncorhynchus mykiss | E–1–o–2 |
| KU878276 | IHNV/O.mykiss/I/TV/651/Jul93 | 1993 | Italy | Oncorhynchus mykiss | E–1–ah–1 |
| KU878277 | IHNV/O.mykiss/I/UD/155/Feb94 | 1994 | Italy | Oncorhynchus mykiss | E–1–l–1 |
| KU878278 | IHNV/O.mykiss/I/TV/148/Mar94 | 1994 | Italy | Oncorhynchus mykiss | E–1–n–1 |
| KU878279 | IHNV/O.mykiss/I/UD/191/Mar94 | 1994 | Italy | Oncorhynchus mykiss | E–1–y–2 |
| KU878280 | IHNV/O.mykiss/I/VI/71/Feb95 | 1995 | Italy | Oncorhynchus mykiss | E–1–h–1 |
| KU878281 | IHNV/O.mykiss/I/TV/576/Oct95 | 1995 | Italy | Oncorhynchus mykiss | E–1–v–2 |
| KU878282 | IHNV/O.mykiss/I/TN/578/Oct95 | 1995 | Italy | Oncorhynchus mykiss | E–1–v–1 |
| KU878283 | IHNV/O.mykiss/I/TN/40/Jan97 | 1997 | Italy | Oncorhynchus mykiss | E–1–y–1 |
| KU878284 | IHNV/O.mykiss/I/UD/83/Feb97 | 1997 | Italy | Oncorhynchus mykiss | E–1–o–1 |
| KU878285 | IHNV/O.mykiss/I/TV/131/Mar97 | 1997 | Italy | Oncorhynchus mykiss | E–2–c–1 |
| KU878286 | IHNV/O.mykiss/I/PN/177/Mar97 | 1997 | Italy | Oncorhynchus mykiss | E–1–g–3 |
| KU878287 | IHNV/O.mykiss/I/TN/459/Sep97 | 1997 | Italy | Oncorhynchus mykiss | E–1–ac–1 |
| KU878288 | IHNV/O.mykiss/I/PN/466/Oct97 | 1997 | Italy | Oncorhynchus mykiss | E–1–aa–1 |
| KU878289 | IHNV/O.mykiss/I/VI/519/Oct97 | 1997 | Italy | Oncorhynchus mykiss | E–1–s–1 |
| KU878290 | IHNV/O.mykiss/I/TN/528/Oct97 | 1997 | Italy | Oncorhynchus mykiss | E–1–ab–4 |
| KU878291 | IHNV/O.mykiss/I/TN/531/Oct97 | 1997 | Italy | Oncorhynchus mykiss | E–1–ab–2 |
| KU878292 | IHNV/O.mykiss/I/TV/603/Nov97 | 1997 | Italy | Oncorhynchus mykiss | E–2–a–13 |
| KU878293 | IHNV/O.mykiss/I/TV/608/Nov97 | 1997 | Italy | Oncorhynchus mykiss | E–2–b–3 |
| KU878294 | IHNV/O.mykiss/I/TV/541/Dec97 | 1997 | Italy | Oncorhynchus mykiss | E–2–a–53 |
| KU878295 | IHNV/O.mykiss/I/PN/686/Dec97 | 1997 | Italy | Oncorhynchus mykiss | E–2–a–9 |
| KU878296 | IHNV/O.mykiss/I/TV/9/Jan98 | 1998 | Italy | Oncorhynchus mykiss | E–2–a–8 |
| KU878297 | IHNV/O.mykiss/I/UD/261/Mar98 | 1998 | Italy | Oncorhynchus mykiss | E–1–z–2 |
| KU878298 | IHNV/O.mykiss/I/UD/592/Oct98 | 1998 | Italy | Oncorhynchus mykiss | E–2–a–14 |
| KU878299 | IHNV/O.mykiss/I/UD/593/Oct98 | 1998 | Italy | Oncorhynchus mykiss | E–2–b–2 |
| KU878300 | IHNV/O.mykiss/I/PN/748/Dec98 | 1998 | Italy | Oncorhynchus mykiss | E–2–c–2 |
| KU878301 | IHNV/O.mykiss/I/TV/343/May99 | 1999 | Italy | Oncorhynchus mykiss | E–1–aa–3 |
| KU878302 | IHNV/O.mykiss/I/TN/673/Dec99 | 1999 | Italy | Oncorhynchus mykiss | E–1–t–1 |
| KU878303 | IHNV/O.mykiss/I/TV/8/Jan00 | 2000 | Italy | Oncorhynchus mykiss | E–1–aa–2 |
| KU878304 | IHNV/O.mykiss/I/UD/63/Feb00 | 2000 | Italy | Oncorhynchus mykiss | E–2–b–4 |
| KU878305 | IHNV/O.mykiss/I/TV/173/Apr00 | 2000 | Italy | Oncorhynchus mykiss | E–2–a–52 |
| KU878306 | IHNV/O.mykiss/I/PN/202/Apr00 | 2000 | Italy | Oncorhynchus mykiss | E–1–z–1 |
| KU878308 | IHNV/O.mykiss/I/TN/431/Oct00 | 2000 | Italy | Oncorhynchus mykiss | E–1–v–3 |
| KU878309 | IHNV/O.mykiss/I/TV/541/Dec00 | 2000 | Italy | Oncorhynchus mykiss | E–2–a–21 |
| KU878310 | IHNV/O.mykiss/I/VE/23/Jan01 | 2001 | Italy | Oncorhynchus mykiss | E–2–a–16 |
| KU878311 | IHNV/O.mykiss/I/TN/6/Jan01 | 2001 | Italy | Oncorhynchus mykiss | E–1–t–2 |
| KU878314 | IHNV/O.mykiss/I/TN/198/May01 | 2001 | Italy | Oncorhynchus mykiss | E–1–v–3 |
| KU878315 | IHNV/O.mykiss/I/VE/204/May01 | 2001 | Italy | Oncorhynchus mykiss | E–2–a–54 |
| KU878316 | IHNV/O.mykiss/I/VI/272/Jun01 | 2001 | Italy | Oncorhynchus mykiss | E–1–s–3/4 |
| KU878316 | IHNV/O.mykiss/I/VI/272/Jun01 | 2001 | Italy | Oncorhynchus mykiss | E–2–a–15 |
| KU878317 | IHNV/O.mykiss/I/VI/372/Oct01 | 2001 | Italy | Oncorhynchus mykiss | E–1–p–6 |
| KU878318 | IHNV/O.mykiss/I/TV/3/Dec02 | 2002 | Italy | Oncorhynchus mykiss | E–1–s–2 |
| KU878319 | IHNV/O.mykiss/I/VI/78/Mar02 | 2002 | Italy | Oncorhynchus mykiss | E–1–v–5 |
| KU878320 | IHNV/S.fontinalis/I/TN/100/Mar02 | 2002 | Italy | Salvelinus fontinalis | E–1–v–6 |
| KU878321 | IHNV/O.mykiss/I/TN/99/Mar02 | 2002 | Italy | Oncorhynchus mykiss | E–1–v–4 |
| KU878322 | IHNV/O.mykiss/I/TN/150/Apr02 | 2002 | Italy | Oncorhynchus mykiss | E–1–v–7 |
| KU878323 | IHNV/O.mykiss/I/UD/185/Apr02 | 2002 | Italy | Oncorhynchus mykiss | E–1–r–1 |
| KU878324 | IHNV/O.mykiss/I/UD/194/May02 | 2002 | Italy | Oncorhynchus mykiss | E–2–a–6 |
| KU878325 | IHNV/O.mykiss/I/TV/299/Jun03 | 2003 | Italy | Oncorhynchus mykiss | E–1–t–3 |
| KU878326 | IHNV/O.mykiss/I/TN/304/Jun03 | 2003 | Italy | Oncorhynchus mykiss | E–2–c–3 |
| KU878327 | IHNV/O.mykiss/I/TV/523/Nov03 | 2003 | Italy | Oncorhynchus mykiss | E–2–c–3 |
| KU878328 | IHNV/O.mykiss/I/TV/525/Nov03 | 2003 | Italy | Oncorhynchus mykiss | E–1–p–1 |
| KU878329 | IHNV/O.mykiss/I/TV/13/Jan04 | 2004 | Italy | Oncorhynchus mykiss | E–1–p–7 |
| KU878332 | IHNV/O.mykiss/I/TV/299/Aug04 | 2004 | Italy | Oncorhynchus mykiss | E–2–c–13 |
| KU878333 | IHNV/O.mykiss/I/VE/323/Sep04 | 2004 | Italy | Oncorhynchus mykiss | E–2–c–14 |
| FJ711517 | I742 | 2005 | Italy | Oncorhynchus mykiss | E–1–p–1 |
| FJ711516 | I687 | 2005 | Italy | Oncorhynchus mykiss | E–2–a–1 |
| FJ711513 | I273 | 2005 | Italy | Oncorhynchus mykiss | E–2–c–6 |
| FJ711510 | I166 | 2005 | Italy | Oncorhynchus mykiss | E–1–s–5 |
| KU878334 | IHNV/O.mykiss/I/TV/107/Mar05 | 2005 | Italy | Oncorhynchus mykiss | E–2–a–10 |
| KU878335 | IHNV/O.mykiss/I/VI/166/Apr05 | 2005 | Italy | Oncorhynchus mykiss | E–1–s–5 |
| KU878336 | IHNV/O.mykiss/I/TV/200/Apr05 | 2005 | Italy | Oncorhynchus mykiss | E–1–r–2 |
| KU878337 | IHNV/O.mykiss/I/UD/273/May05 | 2005 | Italy | Oncorhynchus mykiss | E–2–c–5 |
| KU878339 | IHNV/O.mykiss/I/TV/459/Sep05 | 2005 | Italy | Oncorhynchus mykiss | E–2–c–7 |
| KU878340 | IHNV/O.mykiss/I/UD/594/Nov05 | 2005 | Italy | Oncorhynchus mykiss | E–2–b–6 |
| KU878341 | IHNV/O.mykiss/I/VI/687/Dec05 | 2005 | Italy | Oncorhynchus mykiss | E–2–a–2 |
| KU878342 | IHNV/O.mykiss/I/TV/742/Dec05 | 2005 | Italy | Oncorhynchus mykiss | E–1–p–2 |
| KU878343 | IHNV/O.mykiss/I/TV/389/Nov06 | 2006 | Italy | Oncorhynchus mykiss | E–1–p–3 |
| KU878344 | IHNV/O.mykiss/I/VI/409/Nov06 | 2006 | Italy | Oncorhynchus mykiss | E–2–c–15 |
| FJ711515 | I459-05 | 2005 | Italy | Oncorhynchus mykiss | E–2–c–8 |
| FJ711514 | I389-06 | 2006 | Italy | Oncorhynchus mykiss | E–1–p–5 |
| FJ711512 | I223-06 | 2006 | Italy | Oncorhynchus mykiss | E–2–c–4 |
| FJ711511 | I208-06 | 2006 | Italy | Oncorhynchus mykiss | E–1–r–3 |
| KU878346 | IHNV/O.mykiss/I/TV/100/Apr07 | 2007 | Italy | Oncorhynchus mykiss | E–1–aa–5 |
| KU878346 | IHNV/O.mykiss/I/TV/100/Apr07 | 2007 | Italy | Oncorhynchus mykiss | E–1–aa–4 |
| KU878347 | IHNV/S.trutta/I/TN/216/Jun07 | 2007 | Italy | Salmo trutta | E–1–r–7 |
| KU878349 | IHNV/O.mykiss/I/VI/246/Jun07 | 2007 | Italy | Oncorhynchus mykiss | E–2–c–16 |
| KU878350 | IHNV/O.mykiss/I/TV/234/May08 | 2008 | Italy | Oncorhynchus mykiss | E–2–a–46 |
| KU878351 | IHNV/O.mykiss/I/TV/455/Oct08 | 2008 | Italy | Oncorhynchus mykiss | E–2–c–9 |
| KU878352 | IHNV/O.mykiss/I/TV/459/Oct08 | 2008 | Italy | Oncorhynchus mykiss | E–1–p–4 |
| KU878353 | IHNV/O.mykiss/I/TV/77/Mar09 | 2009 | Italy | Oncorhynchus mykiss | E–1–r–4 |
| KU878354 | IHNV/O.mykiss/I/VR/2/Jan11 | 2011 | Italy | Oncorhynchus mykiss | E–2–a–3 |
| KU878355 | IHNV/O.mykiss/I/PD/291/Aug11 | 2011 | Italy | Oncorhynchus mykiss | E–2–a–7 |
| KU878356 | IHNV/O.mykiss/I/TN/534/Dec11 | 2011 | Italy | Oncorhynchus mykiss | E–1–t–4 |
| KU878357 | IHNV/O.mykiss/I/TV/21/Jan12 | 2012 | Italy | Oncorhynchus mykiss | E–1–r–5 |
| KU878358 | IHNV/O.mykiss/I/TN/103/Feb12 | 2012 | Italy | Oncorhynchus mykiss | E–1–t–4 |
| KU878359 | IHNV/O.mykiss/I/TV/158/May13 | 2013 | Italy | Oncorhynchus mykiss | E–1–r–11 |
| KU878360 | IHNV/O.mykiss/I/TV/206/May13 | 2013 | Italy | Oncorhynchus mykiss | E–1–r–11 |
| KU878361 | IHNV/O.mykiss/I/TN/238/Jun13 | 2013 | Italy | Oncorhynchus mykiss | E–1–r–11 |
|  |  |  |  |  |  |
| HG933992 | E_I12-08NL | 2008 | Netherlands | Oncorhynchus mykiss | E–2–a–24 |
| HG933993 | E_I13-08NL | 2008 | Netherlands | Oncorhynchus mykiss | E–2–a–25 |
| HG933994 | E_I08-09NL | 2009 | Netherlands | Oncorhynchus mykiss | E–2–a–17 |
| HG933997 | E_I15-11NL | 2011 | Netherlands | Oncorhynchus mykiss | E–2–a–17 |
| HG933995 | E_I13-11NL | 2011 | Netherlands | Oncorhynchus mykiss | E–2–a–19 |
| HG933998 | E_I16-11NL | 2011 | Netherlands | Oncorhynchus mykiss | E–2–a–19 |
| HG933996 | E_I14-11NL | 2011 | Netherlands | Oncorhynchus mykiss | E–2–a–18 |
| HG933999 | E_I17-11NL | 2011 | Netherlands | Oncorhynchus mykiss | E–2–a–20 |
| HG934000 | E_I18-11NL | 2011 | Netherlands | Oncorhynchus mykiss | E–2–a–20 |
| HG934001 | E_I19-11NL | 2011 | Netherlands | Oncorhynchus mykiss | E–2–a–20 |
| HG934002 | E_I20-11NL | 2011 | Netherlands | Oncorhynchus mykiss | E–2–a–20 |
| HG934003 | E_I21-11NL | 2011 | Netherlands | Oncorhynchus mykiss | E–2–a–20 |
|  |  |  |  |  |  |
| LN897479 | E_CH14-93 | 1993 | Switzerland | Oncorhynchus mykiss | E–1–ak–2 |
| EU676196 | CH29-96 | 1996 | Switzerland | Oncorhynchus mykiss | E–1–ak–1 |
| LN897480 | E_CH124-97 | 1997 | Switzerland | Oncorhynchus mykiss | E–1–af–1 |
| LN897481 | E_CH213-97 | 1997 | Switzerland | Oncorhynchus mykiss | E–1–af–1 |
| LN897482 | E_CH234-97 | 1997 | Switzerland | Oncorhynchus mykiss | E–1–af–1 |
| LN897483 | E_CH302-97 | 1997 | Switzerland | Oncorhynchus mykiss | E–1–af–1 |
| LN897484 | E_CH312-97 | 1997 | Switzerland | Oncorhynchus mykiss | E–1–af–2 |
| LN897485 | E_CH350-97 | 1997 | Switzerland | Oncorhynchus mykiss | E–1–af–2 |
| EU676199 | CH308-97 | 1997 | Switzerland | Oncorhynchus mykiss | E–1–af–8 |
| LN897486 | E_CH12-98 | 1998 | Switzerland | Oncorhynchus mykiss | E–1–af–1 |
| EU676198 | CH247-00 | 2000 | Switzerland | Oncorhynchus mykiss | E–1–af–3 |
| LN897487 | E_CH88-02 | 2002 | Switzerland | Oncorhynchus mykiss | E–2–a–22 |
| EU676197 | CH118-02 | 2002 | Switzerland | Oncorhynchus mykiss | E–1–aj–1 |
| LN897488 | E_I01-04CH | 2004 | Switzerland | Oncorhynchus mykiss | E–1–v–2 |
| LN897489 | E_I02-04CH | 2004 | Switzerland | Oncorhynchus mykiss | E–1–v–2 |
| LN897490 | E_I03-04CH | 2004 | Switzerland | Oncorhynchus mykiss | E–1–v–2 |
| LN897491 | E_I04-10CH | 2010 | Switzerland | Oncorhynchus mykiss | E–1–r–8 |
| LN897492 | E_I08-10CH | 2010 | Switzerland | Oncorhynchus mykiss | E–1–r–8 |
| LN897493 | E_I09-10CH | 2010 | Switzerland | Oncorhynchus mykiss | E–1–r–8 |
| LN897494 | E_I08-12CH | 2012 | Switzerland | Oncorhynchus mykiss | E–1–aj–3 |
| LN897495 | E_I12-13CH | 2013 | Switzerland | Oncorhynchus mykiss | E–2–a–47 |
| LN897496 | E_I13-13CH | 2013 | Switzerland | Oncorhynchus mykiss | E–2–a–5 |
| LN897497 | E_I22-14CH | 2014 | Switzerland | Oncorhynchus mykiss | E–1–ak–4 |
| LN897499 | E_I24-14CH | 2014 | Switzerland | Oncorhynchus mykiss | E–1–ak–4 |
| LN897498 | E_I23-14CH | 2014 | Switzerland | Oncorhynchus mykiss | E–1–ak–3 |
|  |  |  |  |  |  |
| AY331657 | D332-92 | 1992 | Germany | Oncorhynchus mykiss | E–1–ab–1 |
| LN897500 | I01-93nw | 1993 | Germany | Oncorhynchus mykiss | E–1–w–1 |
| EU676209 | Dau832-94 | 1994 | Germany | Oncorhynchus mykiss | E–1–c–1 |
| AY331661 | s832/94 | 1994 | Germany | Oncorhynchus mykiss | E–1–c–1 |
| AY331662 | Fs30/95 | 1995 | Germany | Oncorhynchus mykiss | E–1–d–1 |
| AY331663 | Fs42/95 | 1995 | Germany | Oncorhynchus mykiss | E–1–g–1 |
| AY331664 | Fs62/95 | 1995 | Germany | Oncorhynchus mykiss | E–1–g–1 |
| EU676208 | Dau819-96 | 1996 | Germany | Oncorhynchus mykiss | E–1–q–1 |
| EU676210 | Dau1036-96 | 1996 | Germany | Oncorhynchus mykiss | E–1–g–4 |
| EU676232 | Dfv47-7-96 | 1996 | Germany | Oncorhynchus mykiss | E–1–g–2 |
| EU676217 | Dfr100-96 | 1996 | Germany | Oncorhynchus mykiss | E–1–f–1 |
| AY331666 | FsVi100/96 | 1996 | Germany | Oncorhynchus mykiss | E–1–f–1 |
| EU676211 | Dau1573-97 | 1997 | Germany | Oncorhynchus mykiss | E–1–e–1 |
| EU676200 | Dau26-97 | 1997 | Germany | Oncorhynchus mykiss | E–1–l–2 |
| EU676202 | Dau32-97 | 1997 | Germany | Oncorhynchus mykiss | E–1–g–3 |
| EU676203 | Dau37-97 | 1997 | Germany | Oncorhynchus mykiss | E–1–ab–3 |
| EU676204 | Dau55-98 | 1998 | Germany | Oncorhynchus mykiss | E–1–m–1 |
| EU676205 | Dau64-98 | 1998 | Germany | Oncorhynchus mykiss | E–1–af–5 |
| LN897501 | E_ihn8-98 | 1998 | Germany | Oncorhynchus mykiss | E–1–af–4 |
| AY331658 | Dfs13-98 | 1998 | Germany | Anguilla anguilla* | E–1–w–1 |
| LN897502 | E_I01-99nw | 1999 | Germany | Oncorhynchus mykiss | E–2–b–1 |
| AY331660 | Fs8/99 | 1999 | Germany | Oncorhynchus mykiss | E–1–af–4 |
| LN897503 | E_I02-99nw | 1999 | Germany | Oncorhynchus mykiss | E–1–af–6 |
| EU676206 | Dau373-01 | 2001 | Germany | Oncorhynchus mykiss | E–1–ag–5 |
| EU676233 | Dns28-02 | 2002 | Germany | Oncorhynchus mykiss | E–1–af–7 |
| AY331659 | Fs28-02 | 2002 | Germany | Oncorhynchus mykiss | E–1–af–7 |
| EU676219 | Dfr864-03 | 2003 | Germany | Oncorhynchus mykiss | E–2–c–23 |
| EU676220 | Dfr946-03 | 2003 | Germany | Oncorhynchus mykiss | E–2–c–22 |
| EU676228 | Dwb42-03 | 2003 | Germany | Oncorhynchus mykiss | E–1–v–8 |
| EU676223 | Dfr1019-03 | 2003 | Germany | Oncorhynchus mykiss | E–2–c–25 |
| EU676231 | Dfr394-04 | 2004 | Germany | Oncorhynchus mykiss | E–2–c–24 |
| EU676207 | Dau688-04 | 2004 | Germany | Oncorhynchus mykiss | E–1–aj–2 |
| EU676214 | Dau4241-04 | 2004 | Germany | Oncorhynchus mykiss | E–2–b–5 |
| EU676215 | De2305-05 | 2005 | Germany | Oncorhynchus mykiss | E–2–c–29 |
| EU676216 | Df1963-05 | 2005 | Germany | Oncorhynchus mykiss | E–2–c–28 |
| EU676213 | Dau2185-05 | 2005 | Germany | Oncorhynchus mykiss | E–2–c–31 |
| EU676221 | Dfr951-05 | 2005 | Germany | Oncorhynchus mykiss | E–2–c–31 |
| EU676222 | Dfr1013-05 | 2005 | Germany | Oncorhynchus mykiss | E–2–c–30 |
| EU676227 | Dw1973-05 | 2005 | Germany | Oncorhynchus mykiss | E–2–c–26 |
| EU676235 | Ds1831-05 | 2005 | Germany | Oncorhynchus mykiss | E–2–c–26 |
| EU676236 | Ds2162-05 | 2005 | Germany | Oncorhynchus mykiss | E–2–c–26 |
| EU676226 | Dswego | 2006 | Germany | Oncorhynchus mykiss | E–1–x–2 |
| LN897506 | fr2866-06 | 2006 | Germany | Oncorhynchus mykiss | E–2–c–31 |
| LN897504 | fr34a-06 | 2006 | Germany | Oncorhynchus mykiss | E–1–x–6 |
| LN897505 | fr34b-06 | 2006 | Germany | Oncorhynchus mykiss | E–1–x–3 |
| EU676225 | Dstgfv106-2-06 | 2006 | Germany | Oncorhynchus mykiss | E–1–x–4 |
| EU676224 | stgfv86-06 | 2006 | Germany | Oncorhynchus mykiss | E–1–x–3 |
| EU676212 | Dau1906-07 | 2007 | Germany | Oncorhynchus mykiss | E–2–c–27 |
| EU676218 | Dfr858-07 | 2007 | Germany | Oncorhynchus mykiss | E–2–c–34 |
| EU676234 | Dnsfv234-07 | 2007 | Germany | Oncorhynchus mykiss | E–2–a–23 |
| HG933970 | E_I01-07th | 2007 | Germany | Oncorhynchus mykiss | E–2–a–49 |
| HG933971 | E_Ibi1-1189-7 | 2007 | Germany | Oncorhynchus mykiss | E–2–a–48 |
| LN897507 | E_I01-08bw | 2008 | Germany | Oncorhynchus mykiss | E–2–c–40 |
| LN897512 | E_I06-08bw | 2008 | Germany | Oncorhynchus mykiss | E–2–c–40 |
| LN897513 | E_I07-08bw | 2008 | Germany | Oncorhynchus mykiss | E–2–c–40 |
| LN897508 | E_I02-08bw | 2008 | Germany | Oncorhynchus mykiss | E–2–c–41 |
| LN897509 | E_I03-08bw | 2008 | Germany | Oncorhynchus mykiss | E–2–c–41 |
| LN897515 | E_I11-08rp | 2008 | Germany | Oncorhynchus mykiss | E–2–c–19 |
| LN897516 | E_I12-08by | 2008 | Germany | Oncorhynchus mykiss | E–2–c–10 |
| HG933972 | E_I13-08by | 2008 | Germany | Oncorhynchus mykiss | E–2–a–50 |
| LN897510 | E_I04-08bw | 2008 | Germany | Oncorhynchus mykiss | E–2–c–41 |
| LN897511 | E_I05-08bw | 2008 | Germany | Oncorhynchus mykiss | E–2–c–41 |
| LN897514 | E_I08-08bw | 2008 | Germany | Oncorhynchus mykiss | E–2–c–41/42 |
| LN897517 | E_I02-09sx | 2009 | Germany | Oncorhynchus mykiss | E–1–ai–1 |
| LN897519 | E_I06-09by | 2009 | Germany | Oncorhynchus mykiss | E–2–c–44 |
| HG933973 | E_I04-09by | 2009 | Germany | Oncorhynchus mykiss | E–2–a–51 |
| LN897518 | E_I05-09by | 2009 | Germany | Oncorhynchus mykiss | E–2–c–12 |
| LN897520 | E_I07-09by | 2009 | Germany | Oncorhynchus mykiss | E–2–c–43 |
| LN897524 | E_I07-10ns | 2010 | Germany | Oncorhynchus mykiss | E–2–c–18 |
| LN897523 | E_I03-10bw | 2010 | Germany | Oncorhynchus mykiss | E–2–c–33 |
| LN897521 | E_I01-10ns | 2010 | Germany | Oncorhynchus mykiss | E–2–c–17 |
| LN897522 | E_I02-10sx | 2010 | Germany | Oncorhynchus mykiss | E–2–c–20 |
| LN897525 | E_I08-10by | 2010 | Germany | Oncorhynchus mykiss | E–2–c–11 |
| LN897528 | E_I08-11sx | 2011 | Germany | Oncorhynchus mykiss | E–2–a–11 |
| HG933974 | E_I02-11ns | 2011 | Germany | Oncorhynchus mykiss | E–2–a–27 |
| LN897526 | E_I01-11sx | 2011 | Germany | Oncorhynchus mykiss | E–2–a–12 |
| HG933975 | E_I03-11bb | 2011 | Germany | Oncorhynchus mykiss | E–2–a–28 |
| HG933977 | E_I06-11th | 2011 | Germany | Oncorhynchus mykiss | E–2–a–29 |
| HG933980 | E_I10-11nw | 2011 | Germany | Oncorhynchus mykiss | E–2–a–26 |
| HG933981 | E_I11-11nw | 2011 | Germany | Oncorhynchus mykiss | E–2–a–26 |
| LN897529 | E_I12-11bw | 2011 | Germany | Oncorhynchus mykiss | E–1–x–1 |
| HG933978 | E_I07-11sx | 2011 | Germany | Oncorhynchus mykiss | E–2–a–31 |
| HG933979 | E_I09-11sx | 2011 | Germany | Oncorhynchus mykiss | E–2–a–32 |
| LN897527 | E_I05-11th | 2011 | Germany | Oncorhynchus mykiss | E–2–c–21 |
| HG933976 | E_I04-11bb | 2011 | Germany | Oncorhynchus mykiss | E–2–a–28 |
| HG933983 | E_I03-12by | 2012 | Germany | Oncorhynchus mykiss | E–2–a–4 |
| HG933984 | E_I04-12by | 2012 | Germany | Oncorhynchus mykiss | E–2–a–4 |
| HG933982 | E_I01-12sa | 2012 | Germany | Oncorhynchus mykiss | E–2–a–30 |
| HG933985 | E_I05-12by | 2012 | Germany | Oncorhynchus mykiss | E–2–a–4 |
| LN897530 | E_I02-12bw | 2012 | Germany | Oncorhynchus mykiss | E–1–g–5 |
| HG933986 | E_I01-13sa | 2013 | Germany | Oncorhynchus mykiss | E–2–a–36 |
| HG933989 | E_I07-13bw | 2013 | Germany | Oncorhynchus mykiss | E–2–a–36 |
| LN897531 | E_I02-13by | 2013 | Germany | Oncorhynchus mykiss | E–1–r–11 |
| LN897532 | E_I03-13by | 2013 | Germany | Oncorhynchus mykiss | E–1–r–11 |
| HG933987 | E_I05-13bb | 2013 | Germany | Oncorhynchus mykiss | E–2–a–34 |
| LN897534 | E_I11-13bw | 2013 | Germany | Oncorhynchus mykiss | E–2–c–36 |
| HG933988 | E_I06-13bb | 2013 | Germany | Oncorhynchus mykiss | E–2–a–35 |
| HG933990 | E_I08-13sx | 2013 | Germany | Oncorhynchus mykiss | E–2–a–43 |
| LN897533 | E_I04-13by | 2013 | Germany | Oncorhynchus mykiss | E–1–r–11 |
| HG933991 | E_I09-13sx | 2013 | Germany | Oncorhynchus mykiss | E–2–a–37 |
| LN897535 | E_I15-13by | 2013 | Germany | Oncorhynchus mykiss | E–1–r–11 |
| LN897537 | E_I02-14bb | 2014 | Germany | Oncorhynchus mykiss | E–1–r–6 |
| LN897536 | E_I01-14bb | 2014 | Germany | Oncorhynchus mykiss | E–2–a–55 |
| LN897542 | E_I08-14bb | 2014 | Germany | Oncorhynchus mykiss | E–1–r–6 |
| LN897538 | E_I03-14bw | 2014 | Germany | Oncorhynchus mykiss | E–2–c–37 |
| LN897539 | E_I05-14bw | 2014 | Germany | Oncorhynchus mykiss | E–2–c–37 |
| LN897540 | E_I06-14bw | 2014 | Germany | Oncorhynchus mykiss | E–2–c–37 |
| LN897541 | E_I07-14bw | 2014 | Germany | Oncorhynchus mykiss | E–2–c–37 |
| LN897543 | E_I09-14bw | 2014 | Germany | Oncorhynchus mykiss | E–2–c–35 |
| LN897554 | E_I25-14bw | 2014 | Germany | Oncorhynchus mykiss | E–2–c–35 |
| LN897544 | E_I10-14bw | 2014 | Germany | Oncorhynchus mykiss | E–2–c–39 |
| LN897545 | E_I13-14bw | 2014 | Germany | Oncorhynchus mykiss | E–2–c–38/39 |
| LN897546 | E_I14-14ns | 2014 | Germany | Oncorhynchus mykiss | E–2–a–44 |
| LN897547 | E_I15-14nw | 2014 | Germany | Oncorhynchus mykiss | E–2–a–44 |
| LN897548 | E_I16-14nw | 2014 | Germany | Oncorhynchus mykiss | E–2–a–44 |
| LN897549 | E_I17-14ns | 2014 | Germany | Oncorhynchus mykiss | E–2–a–44 |
| LN897555 | E_I26-14bw | 2014 | Germany | Oncorhynchus mykiss | E–2–a–44 |
| LN897550 | E_I18-14ns | 2014 | Germany | Oncorhynchus mykiss | E–2–a–38 |
| LN897551 | E_I19-14ns | 2014 | Germany | Oncorhynchus mykiss | E–2–a–38 |
| LN897552 | E_I20-14by | 2014 | Germany | Oncorhynchus mykiss | E–2–c–45 |
| LN897553 | E_I21-14by | 2014 | Germany | Oncorhynchus mykiss | E–2–c–45 |
| LN897556 | E_I27-14bw | 2014 | Germany | Oncorhynchus mykiss | E–2–a–45 |
| LN897557 | E_I36-14bw | 2014 | Germany | Oncorhynchus mykiss | E–1–r–12 |
| LN897558 | E_I37-14bw | 2014 | Germany | Oncorhynchus mykiss | E–1–r–12 |
| LN897560 | E_I43-14bw | 2014 | Germany | Oncorhynchus mykiss | E–1–r–12 |
| LN897559 | E_I42-14bw | 2014 | Germany | Oncorhynchus mykiss | E–1–r–9 |
| LN897561 | E_I44-14by | 2014 | Germany | Oncorhynchus mykiss | E–1–r–10 |
| LN897575 | E_I15-15bw | 2015 | Germany | Oncorhynchus mykiss | E–2–a–39 |
| LN897574 | E_I14-15bw | 2015 | Germany | Oncorhynchus mykiss | E–2–a–39 |
| LN897573 | E_I13-15bw | 2015 | Germany | Oncorhynchus mykiss | E–2–a–39 |
| LN897572 | E_I12-15bw | 2015 | Germany | Oncorhynchus mykiss | E–2–a–40 |
| LN897571 | E_I11-15bw | 2015 | Germany | Oncorhynchus mykiss | E–2–a–41 |
| LN897570 | E_I10-15bw | 2015 | Germany | Oncorhynchus mykiss | E–2–a–40/42 |
| LN897569 | E_I09-15bw | 2015 | Germany | Oncorhynchus mykiss | E–2–a–40 |
| LN897568 | E_I08-15bw | 2015 | Germany | Oncorhynchus mykiss | E–2–a–40 |
| LN897567 | E_I07-15bw | 2015 | Germany | Oncorhynchus mykiss | E–2–a–40 |
| LN897566 | E_I06-15bw | 2015 | Germany | Oncorhynchus mykiss | E–2–a–40 |
| LN897565 | E_I05-15bw | 2015 | Germany | Oncorhynchus mykiss | E–2–a–40 |
| LN897564 | E_I04-15bw | 2015 | Germany | Oncorhynchus mykiss | E–2–a–40 |
| LN897563 | E_I03-15bw | 2015 | Germany | Oncorhynchus mykiss | E–2–a–40 |
| LN897562 | E_I02-15bw | 2015 | Germany | Oncorhynchus mykiss | E–2–a–40 |
|  |  |  |  |  |  |
| L40875 | CST-82 | 1982 | USA | Oncorhynchus mykiss | M |
