# Supplementary material for: Phylogeny of the infectious hematopoietic necrosis virus in European aquaculture
Source: PLoS One. 2017 Sep 8;12(9):e0184490. doi: 10.1371/journal.pone.0184490 (PMC5590938; doi:10.1371/journal.pone.0184490)
Supplement: S2 Table — Primers used for RT-PCR and sequencing of IHNV isolates in the present study. (DOCX) [file pone.0184490.s003.docx]

**S2 Table. Primer sequences.**

Primers used for RT-PCR and sequencing of IHNV isolates in the present study.

| **Name** | **Orientation** | **Nucleotide position** | **Sequence** (5ʹ🡪 3ʹ) |
| --- | --- | --- | --- |
|  |  | (*reference sequence* X18263) |  |
| I2761for | forward | 2761-2781 | 5ʹ-AGC TAC CAG AGG GTC AAA GAC -3ʹ |
| I3194for | forward | 3195-3215 | 5ʹ-AAG GAT CTT CGA CGA TGA GAA -3ʹ |
| I3598for | forward | 3598-3618 | 5ʹ-CAT TGG TCC AAC GTC GTT TGG -3ʹ |
| I4063for | forward | 4063-4083 | 5ʹ-TAT CAC GGG ATG TGC ATG ACG -3ʹ |
| I4653rev | reverse | 4653-4633 | 5ʹ-AGA ACT TCT CTG AGT GCC TCC -3ʹ |
| I4211rev | reverse | 4211-4191 | 5ʹ-GTG TTG TTT CCG TGC AAT CCG -3ʹ |
| I3712rev  I3320rev | reverse  reverse | 3712-3692  3320-3300 | 5ʹ-TTG CCT TCA CGA CTC GAT TGG -3ʹ  5ʹ-CGG TAC AGG ACT TTG TGG ATG -3ʹ |
